# A Double-Blind Placebo-Controlled Trial of Maca Root as Treatment for Antidepressant-Induced Sexual Dysfunction in Women

**DOI:** 10.1155/2015/949036

**Published:** 2015-04-14

**Authors:** Christina M. Dording, Pamela J. Schettler, Elizabeth D. Dalton, Susannah R. Parkin, Rosemary S. W. Walker, Kara B. Fehling, Maurizio Fava, David Mischoulon

**Affiliations:** ^1^Depression Clinical and Research Program, Department of Psychiatry, Massachusetts General Hospital, Boston, MA 02114, USA; ^2^Department of Psychiatry and Behavioral Sciences, Emory University, Atlanta, GA 30322, USA

## Abstract

*Objective*. We sought to demonstrate that maca root may be an effective treatment for antidepressant-induced sexual dysfunction (AISD) in women. *Method*. We conducted a 12-week, double-blind, placebo-controlled trial of maca root (3.0 g/day) in 45 female outpatients (mean age of 41.5 ± 12.5 years) with SSRI/SNRI-induced sexual dysfunction whose depression remitted. Endpoints were improvement in sexual functioning as per the Arizona Sexual Experience Scale (ASEX) and the Massachusetts General Hospital Sexual Function Questionnaire (MGH-SFQ). *Results*. 45 of 57 consented females were randomized, and 42 (30 premenopausal and 12 postmenopausal women) were eligible for a modified intent-to-treat analysis based on having had at least one postmedication visit. Remission rates by the end of treatment were higher for the maca than the placebo group, based on attainment of an ASEX total score ≤ 10 (9.5% for maca versus 4.8% for placebo), attaining an MGH-SFQ score ≤ 12 (30.0% for maca versus 20.0% for placebo) and reaching an MGH-SFQ score ≤ 8 (9.5% for maca versus 5.0% for placebo). Higher remission rates for the maca versus placebo group were associated with postmenopausal status. Maca was well tolerated. *Conclusion*. Maca root may alleviate SSRI-induced sexual dysfunction in postmenopausal women. This trial is registered with NCT00568126.

## 1. Introduction

Antidepressant-induced sexual dysfunction (AISD) is a significant complication in the treatment of patients with mood and anxiety disorders, affecting more than half of patients who are taking selective serotonin reuptake inhibitors (SSRIs) or serotonin-norepinephrine reuptake inhibitors (SNRIs) [1], and has been associated with reduced quality of life, reduced self-esteem, and negative effects on mood and relationships [1]. Sexual dysfunction may affect any phase of the sexual response cycle including libido, arousal, and/or orgasm [2]. Because adequate sexual function is an integral aspect of quality of life, many patients whose depression is in remission as a result of taking antidepressant medication may elect to switch or discontinue their treatment, increasing their risk of complications from their underlying psychiatric disorder [3]. AISD is a major reason for medication discontinuation [4].

In the postmenopausal population there are few treatments available for sexual dysfunction regardless of etiology, and none are approved by the FDA for this indication. There is some evidence to support the use of the testosterone patch, though safety concerns restrict its availability in the US [5]. Bupropion has been studied largely in premenopausal women with sexual dysfunction with modest evidence of efficacy [6, 7]. While a recent study has suggested a benefit from the use of the phosphodiesterase inhibitors in premenopausal women suffering from AISD [8], a recent meta-analysis has demonstrated inconsistent effects of the phosphodiesterase inhibitors in women [9]. Given the limited options for women with AISD, many have turned to complementary and natural treatments in the hope of improving sexual functioning [10]. One such natural agent is maca root (*Lepidium meyenii*).

Maca is a hardy perennial plant cultivated in the Andean Mountains that is traditionally used for both nutritional and fertility-enhancing purposes [11]. Animal studies have demonstrated improvement in sexual behaviors such as increased copulatory attempts due to maca [12–18] and anecdotal evidence certainly supports the use of maca for the treatment of sexual dysfunction in humans [11]. However, few controlled clinical trials in humans have been conducted and those few have generally studied only males. In humans, maca has been shown to increase sperm count and sperm motility [19], as well as increasing sexual desire [20–23]. However the mechanism by which maca exerts its purported aphrodisiac and fertility-enhancing properties is unclear.

In one of the few published studies of maca in women, early postmenopausal women treated with maca were more likely than those who received placebo to show significant decreases in follicle stimulating hormone (FSH) and significant increases in luteinizing hormone (LH) production [24]. In addition, several studies do at least suggest that maca improves menopausal symptoms [20, 24]. For the postmenopausal female population, while the paucity of data precludes any reasonable inference of effectiveness, let alone mechanism of the effect [25], it is quite possible that maca ameliorates postmenopausal symptoms through an androgenic mechanism. Recently, an interesting case report in the British Medical Journal detailed the case of testosterone assay interference by maca, suggesting that maca contains an as of yet unspecified compound with a similar moiety to the human testosterone molecule and that it may be exerting its androgenic effects through actions at the testosterone receptor on target organs without affecting the level of testosterone or gonadotrophins [26]. This may explain why studies in men demonstrate a lack of effect of maca on serum hormone levels [19, 22, 27, 28].

In a prior trial we recently demonstrated that maca treatment may yield improvement in libido in women with AISD [21]. At a dose of 3 grams per day women described greater sexual activity and more enjoyable sexual experiences. In view of the encouraging preliminary findings, we sought to validate the results of our first open-label dose-finding study by conducting a double-blind placebo-controlled trial of maca root in the treatment of AISD in women. An additional aim of the study was to document the safety and tolerability of maca root. Further we intend to explore the hypothesis that the salutary effects of maca on sexual dysfunction derive from an androgenic response.

## 2. Materials and Methods

### 2.1. Subjects

This study screened 57 and included 45 remitted depressed female outpatients aged 18–65 (mean age 41.5 years ± 12.5) who were currently suffering from AISD. Patients were required to be in remission from any depressive or anxiety disorder with a score of 9 or less on the 17-item Hamilton Rating Scale for Depression (HAM-D-17) [29] and a score of 9 or less on the Hamilton Rating Scale for Anxiety (HAM-A) [30] to indicate remission. To be enrolled in the study, patients had to have been taking an SSRI, venlafaxine, or a tri/hetero cyclic antidepressant for the treatment of depression at a stable dose for at least 4 weeks. Additionally, they had to have been suffering from clinically significant arousal dysfunction or orgasmic dysfunction for at least four weeks, and the dysfunction had to have emerged subsequent to the use of the currently prescribed antidepressant (determined by self-report). Lastly, all patients must have been partaking in some form of regular sexual activity (i.e., masturbation, oral sex, intercourse) at least twice monthly prior to antidepressant use and must have been willing to continue sexual activity at least once weekly for the duration of the study.

Patients were excluded if they had been diagnosed with a sexual disorder in the past, were currently receiving another medical or therapeutic form of treatment for their sexual dysfunction, were experiencing sexual dysfunction due to a general underlying medical condition, had experienced recent major relationship changes or turmoil unrelated to the sexual dysfunction, or had any other general health problems or social situations that might have influenced sexual dysfunction or its treatment.

The patients were recruited from December of 2007 through June of 2010 through the Depression Clinical and Research Program in Boston, MA. The Institutional review Board of Massachusetts General Hospital (MGH) approved this study, and all patients gave their written informed consent before study procedures began.

After a diagnostic screening and baseline visit, eligible patients were randomized in a double-blind manner to maca root 1500 mg bid or placebo bid, for 12 weeks. All patients were assessed biweekly using the Massachusetts General Hospital-Sexual Functioning Questionnaire (MGH-SFQ) [31] and the Arizona Sexual Experience Scale (ASEX) [32], both five-item rating scales that evaluate the various areas of sexual function (i.e., arousal, orgasm, and satisfaction). Patients were also assessed for degree and improvement of AISD by the Clinical Global Impression-Severity and Clinical Global Impression-Improvement scales (CGI-S and CGI-I) [33], respectively. To monitor depressive, anxious, and other clinical symptoms, we used the 28-item HAM-D, the 14-item HAM-A, and the Kellner's Symptoms Questionnaire (SQ) [34]. Additionally, side effects, adverse events, and concomitant medications were recorded by the treating psychiatrist at every visit. A nonblind physician had access to the study randomization list in order to provide appropriate follow-up care to patients who dropped out of the study. At the end of the 12 weeks of the study, patients were offered three months of follow-up care.

Blood samples were obtained from all study participants and assayed for estradiol, progesterone, prolactin, and testosterone, in the MGH Clinical Laboratory.

The maca product we used, including the commercial preparation as well as a sample of the source plant obtained from the Peruvian manufacturer, was analyzed and vouchered by botanists at the Harvard University Herbaria and the Massachusetts College of Pharmacy and Health Sciences (MCPHS). Macamide content was determined by liquid chromatography-tandem mass spectrometry (LC-MS) using standard macamides. Our sample consisted of N-benzyl-palmitamide (42.2 *μ*g/g of plant material), N-benzyl-stearamide (10.6 *μ*g/g), N-benzyl-oleamide (16.4 *μ*g/g), N-(methoxy-benzyl)-oleamide (2.4 *μ*g/g), N-benzyl-linoleamide (49.8 *μ*g/g), N-(methoxy-benzyl)-linoleamide (7.3 *μ*g/g), N-benzyl-linoleniamide (53.5 *μ*g/g), N-(methoxy-benzyl)-linoleniamide (4.1 *μ*g/g), and N-(methoxy)-benzylpalmitamide (4.4 *μ*g/g). The macamide concentrations were consistent with those in previously analyzed maca samples.

### 2.2. Statistical Analyses

Analyses were conducted on the basis of a modified intent-to-treat (ITT) sample and consisted of 42 women who completed at least one clinical assessment after a minimum of seven days of treatment. This included 21 women (14 premenopausal and 7 postmenopausal) randomized to treatment with maca and 21 women (16 premenopausal and 5 postmenopausal) randomized to placebo. Analyses were performed for each aggregate treatment arm; subgroup analyses based upon menopausal status were also performed. Significance of change in total ASEX and MGH-SFQ scores between baseline and final assessment were assessed by the paired *t*-test.

Treatment groups were also compared based on the percentage reaching either of two remission thresholds per scale: a total score of 12 (“minimally diminished”) or less and of 8 (“normal”) or less on the MGH-SFQ scale and a total score of 10 (“very strong/very easily/very satisfying”) or less and of 8 or less on the ASEX.

Remission rates for maca versus placebo were compared by means of relative risk (if the number meeting remission in the placebo group was greater than zero) and by odds ratio (with 95% confidence interval). These analyses were repeated for each of the four individual items of the MGH-SFQ assessing interest, arousal, orgasm, and satisfaction. For the item analyses, groups were compared on the number and percentage reaching a final status of “minimally diminished” or better relative to their “normal” functioning (self-rating of 1, 2, or 3).

The paired samples *t*-test was used to assess changes in serum testosterone from baseline to endpoint for each treatment arm. Differences between the two treatment arms and between pre- and postmenopausal women were compared using the independent samples *t*-test.

## 3. Results

Mean change in total ASEX and MGH-SFQ (−4.03 for maca and −3.06 for placebo on the MGH-SFQ and −3.84 for maca and −2.11 for placebo on the ASEX) scores was not significantly different for the maca versus placebo groups, either overall or within premenopausal or postmenopausal subgroups. Remission rates were higher for the maca group than the placebo group for attainment of an ASEX total score of ≤10 (9.5% for maca versus 4.8% for placebo), attaining an MGH-SFQ score of ≤12 (30.0% for maca versus 20.0% for placebo) and reaching an MGH-SFQ score of ≤8 (9.5% for maca versus 5.0% for placebo). No women reached a threshold of 8 or less on their total ASEX score. Results are summarized on Table 1.

When adjusted for menopausal status, the higher remission rates for the overall maca versus placebo group were attributable to the postmenopausal women. Among the very small group of postmenopausal women, remission rates by all three total score criteria were notably higher for maca than for placebo: 14.3% versus 0.0% for ASEX ≤ 10 (Maca/Placebo Odds Ratio (95% CI) = 5.553 (0.104–295.862)); 57.1% versus 20.0% for MGH ≤ 12 (Maca/Placebo Odds Ratio (95% CI) = 5.333 (0.375–75.779)); and 14.3% versus 0.0% for MGH ≤ 8 (Maca/Placebo Odds Ratio (95% CI) = 5.553 (0.104–295.862)). Among premenopausal women, by contrast, remission rates by two measures (ASEX ≤ 10 and MGH-SFQ ≤ 8) were similar for maca and placebo; on the third measure (MGH-SFQ ≤ 12) the remission rate was considerably lower for maca than for placebo. Results are summarized on Tables 1 and 2. Analysis of specific areas of sexual function showed that, among postmenopausal women, maca improved orgasm (MGH-SFQ item 3), while there was no difference between premenopausal women on maca versus placebo for this item. Among premenopausal (but not postmenopausal women), maca improved arousal (MGH- SFQ item 2). Results are summarized in Table 2.

Advancing age correlated significantly with improvement in sexual functioning as measured by the ASEX in the maca but not in the placebo group (*R* = 0.626, *R* squared = 0.392, and *P* = 0.005).

Change in testosterone level from baseline to endpoint did not correlate significantly with improvements in sexual functioning in the placebo group but did correlate significantly in the maca group as measured by the ASEX (*R* = 0.834, *R* squared = 0.95, and *P* = 0.042). Endpoint testosterone levels correlated with improvements in sexual functioning in the maca group alone as measured by the ASEX (*R* = 0.720, *R* squared = 0.518, and *P* = 0.008). The MGH-SFQ trended towards significance with a *P* = 0.057.

Exploratory analyses of the hormones were performed, including estradiol, progesterone, and prolactin (*P* > 0.05 for all comparisons).

### 3.1. Tolerability of Maca

The maca treatment was well tolerated overall. Three subjects discontinued their participation in the study due to adverse events, such as flu-like symptoms and vomiting. In all cases, it was difficult to attribute these symptoms to maca.

## 4. Discussion

This is, to our knowledge, the first double-blind placebo-controlled study to examine the efficacy and safety of maca as a treatment for antidepressant-induced sexual dysfunction in women. It expands on the findings of our first open-label study (in which 85% of the participants were women) that demonstrated notable mean improvement in sexual function, with the most robust and statistically significant improvement in those subjects receiving 3.0 rather than 1.5 grams of maca per day. In that study, maca also resulted in a significant improvement in libido in the ITT sample, particularly in the high dose group.

In this double-blind, placebo-controlled, follow-up study, remission rates of sexual dysfunction by the end of treatment were higher for the maca than the placebo group. This difference seemed to be driven by a cohort of postmenopausal women, though we believe that this difference may be more a function of advanced age in the menopausal group rather than by menopausal status itself, as we also found a correlation of improvement in the ASEX sexual dysfunction scores with advancing age and no correlation with estrogen levels that decline precipitously in the postmenopausal period. Testosterone levels are known to gradually decrease with age in general, declining after age 25 and continuing to do so through menopause [35]. We found a significant correlation between change in testosterone and testosterone level at endpoint with improvement in the ASEX in the maca group alone. Of note is the finding from a previous study that postmenopausal women treated with maca were more likely than those who received placebo to show significant decreases in FSH and significant increases in LH production [24]. We postulate that these changes (in LH and FSH) via a negative feedback loop result in an increased production of androgens that could explain the improvement in sexual functioning.

Our findings on the relationship between maca and testosterone are interesting and may shed light on the mechanisms of maca. Change in testosterone level from baseline to endpoint and endpoint testosterone levels correlated with improvement in sexual function in the maca group alone. These results seem to be inconsistent with previous findings in men that failed to determine a direct androgenic effect of maca. Clearly however these findings are not generalizable to women; in women it is quite possible that maca may have an indirect androgenic effect via a negative feedback loop involving LH/FSH. It is clearly difficult to interpret the testosterone findings given the recent suggestion that maca may actually interfere with the testosterone assay and exert its effect through actions at the testosterone receptor on target organs without affecting the level of testosterone or gonadotrophins.

Our study is limited by a number of factors. The sample size was relatively small. Additionally, we relied on patient self-report in the domain of orgasm, which is subjective and may be subject to bias and inaccuracy. Until recently, available methods for measuring the sex steroids have been dependent on antibody based assays that employ a range of different detection systems including the use of isotopes or chemical signaling molecules that produce chemiluminescence. These assays have become increasingly more sensitive for the measurement of testosterone but are still incapable of providing the proper low-end sensitivity for analyzing testosterone in females' blood specimens. Recent advances in the use of ultrasensitive methods such as mass spectrometry coupled to either gas or liquid chromatography have improved the technology for measuring testosterone and other low concentration sex steroids to the degree that mass spectrometry based methods are now capable of measuring testosterone in normal women and in women with extremely low levels of testosterone [35], and running a more sensitive assay for testosterone in women may have elucidated the mechanism of action in this cohort.

Finally, while maca was well tolerated and safe, it bears mentioning that, due to budgetary restrictions, we did not obtain liver function tests (LFTs) for study participants. Many popular herbal extracts may induce elevation in LFTs and, in some extreme cases, mild liver damage. This could be a concern if higher doses are used. Future investigations of maca will include LFTs before and after treatment.

## 5. Conclusions

The current study provides evidence that there may be factors such as androgen levels that mediate the efficacy of maca treatment. Further research, informed by the results of the current study and specifically designed to focus on endocrinologic measures, is required in order to fully examine maca root as a treatment for women with AISD. We are currently developing a larger follow-up study to examine the efficacy of maca root in women with AISD. The follow-up study we are developing will include a more sensitive assay for the measurement of testosterone in women.

In summary, maca root may alleviate AISD as women age, particularly in the domain of orgasm. A larger study in this population using higher doses of maca is in preparation.

## Figures and Tables

**Table 1:** Subjects reaching four remission criteria, based on both MGH-SFQ^a^ and ASEX^b^, by treatment and menopausal status.

Remission criterion	Menopausal status	Maca		Placebo		Maca/placebo relative risk^d^	Maca/placebo odds ratio (95% CI)^e^	Number needed to treat (NNT)^f^
		*N*	*N* (%)^c^ remitted	*N*	*N* (%)^c^ remitted			
ASEX ≤10	Before	14	1 (7.14)	16	1 (6.25)	1.143	1.154 (0.065–20.342)	112
	After	7	1 (14.29)	5	0 (0.0)	—	5.553 (0.104–295.862)	7
	Total	**21**	**2 (9.52)**	**21**	**1 (4.76)**	**2.000**	**2.105 (0.176–25.172)**	**21**

ASEX ≤8	Before	14	0 (0.0)	16	0 (0.0)	—	—	—
	After	7	0 (0.0)	5	0 (0.0)	—	—	—
	Total	**21**	**0 (0.0)**	**21**	**0 (0.0)**	**—**	**—**	**—**

MGH-SFQ ≤12	Before	13^h^	2 (15.38)	15^i^	4 (26.67)	0.577	0.500 (0.075–3.316)	9 (NNH)^g^
	After	7	4 (57.14)	5	1 (20.00)	2.857	5.333 (0.375–75.779)	2
	Total	**20**	**6 (30.00)**	**20**	**4 (20.00)**	**1.500**	**1.714 (0.400–7.340)**	**10**

MGH-SFQ ≤8	Before	14	1 (7.14)	15^i^	1 (6.67)	1.071	1.077 (0.061–19.047)	210
	After	7	1 (14.29)	5	0 (0.0)	—	5.553 (0.104–295.862)	7
	Total	**21**	**2 (9.52)**	**20**	**1 (5.00)**	**1.905**	**2.000 (0.167–23.962)**	**23**

^a^MGH-SFQ: Massachusetts General Hospital-Sexual Functioning Questionnaire.

^b^ASEX: Arizona Sexual Experience Scale.

^c^Percentages are shown to 2 decimal places, for greater accuracy in computing relative risk, odds ratio, and NNT.

^d^Relative risk is calculated by maca % remitted/placebo % remitted.

^e^An odds ratio is calculated by (maca % remitted/% nonremitted)/(placebo % remitted/% nonremitted). Odd ratios and their confidence intervals were obtained by entering the cell counts (number remitted/not) into a calculator available at http://www.hutchon.net/ConfidOR.htm. For values with a zero in any cell, an on-line calculator using the null hypothesis (http://www.hutchon.net/ConfidORnulhypo.htm) was used; caution is urged in interpreting these values. All odds ratio and 95% confidence interval values involving nonzero cells were confirmed against a second on-line calculator available at http://faculty.vassar.edu/lowry/odds2x2.html. The odds ratio is mathematically important but difficult to interpret. For that reason, number needed to treat (NNT) is often included as a more clinically interpretable statistic (see below).

^f^Number needed to treat (NNT) is calculated by 1/(maca % remitters − placebo % remitters). (The conservative convention is to round up, for anything but an even whole number.) Because the odds ratio is difficult to interpret, NNT is becoming the accepted “effect size” measure for binary outcomes. It is directly interpretable as the number of people you need to treat with maca in order to have one more remitter than in the placebo group, that is, the number needed to treat to have one more person reaching the benefit criterion. A convention is emerging, where 5 is considered a “good” number, but this can vary depending on the risk/benefit and cost associated with the active treatment.

^g^Because the remission rate is lower for maca than for placebo, results are presented as the number needed to harm (NNH), which is interpreted as the number of people you need to treat with maca in order to have one more failure to remit than in the placebo group.

^h^One subject is not included for either MGH-SFQ remission criterion because baseline MGH-SFQ total = 8.

^i^One subject is not included for MGH-SFQ ≤12 remission criterion because baseline MGH-SFq total = 12.

**Table 2:** Subjects reaching improvement threshold on MGH-SFQ^a^ items by the last visit, by treatment and menopausal status (based on the number of subjects above the threshold at baseline).

Item rated “minimally diminished” or better (1–3) by last visit	Menopausal status	Maca		Placebo		Maca/placebo relative risk^d^	Maca/placebo odds ratio (95% CI)^e^	Number needed to treat (NNT)^f^
		*N* ^b^	*N* (%)^c^ remitted	*N* ^b^	*N* (%)^c^ remitted			
Item 1	Before	11	3 (27.27)	14	5 (35.71)	0.764	0.675 (0.121–3.767)	12 (NNH)^g^
Interest	After	5	2 (40.00)	4	2 (50.00)	0.800	0.667 (0.047–9.473)	10 (NNH)^g^

Item 2	Before	13	8 (61.54)	15	4 (26.67)	2.307	4.400 (0.889–21.781)	3
Arousal	After	5	2 (40.00)	4	2 (50.00)	0.800	0.667 (0.047–9.473)	10 (NNH)^g^

Item 3	Before	12	4 (33.33)	15	5 (33.33)	1.000	1.000 (0.200–5.004)	—
Orgasm	After	7	4 (50.00)	5	0 (0.0)	—	10.561 (1.027–108.641)	2

Item 4	Before	13	5 (38.46)	15	5 (33.33)	1.154	1.250 (0.265–5.886)	19
Satisfaction	After	6	3 (50.00)	4	2 (50.00)	1.000	1.000 (0.080–12.558)	—

^a^MGH-SFQ: Massachusetts General Hospital-Sexual Functioning Questionnaire.

^b^ *N*: the number of subjects who were above the threshold at baseline.

^c^Percentages are shown to 2 decimal places, for greater accuracy in computing relative risk, odds ratio, and NNT.

^d^Relative risk is calculated by maca %/placebo % reaching the benefit threshold.

^e^See footnote e, Table 1.

^f^See footnote f, Table 1.

^g^See footnote g, Table 1.
